# Progress of linking gut microbiota and musculoskeletal health: casualty, mechanisms, and translational values

**DOI:** 10.1080/19490976.2023.2263207

**Published:** 2023-10-06

**Authors:** Yu Wang, Yusheng Li, Lin Bo, Enyuan Zhou, Yanyan Chen, Shinen Naranmandakh, Wenqing Xie, Qin Ru, Lin Chen, Zhaohua Zhu, Changhai Ding, Yuxiang Wu

**Affiliations:** a Department of Health and Kinesiology, School of Physical Education, Jianghan University, Wuhan, China; b Department of Orthopedics, Xiangya Hospital, Central South University, Changsha, China; c National Clinical Research Center for Geriatric Disorders, Xiangya Hospital, Central South University, Changsha, China; d Department of Rheumatology, The Second Affiliated Hospital of Soochow University, Suzhou, Jiangsu, China; e School of Arts and Sciences, National University of Mongolia, Ulaanbaatar, Mongolia; f Clinical Research Centre, Orthopedic Centre, Zhujiang Hospital, Southern Medical University, Guangzhou, China; g Department of Rheumatology, Guangzhou First People’s Hospital, School of Medicine, South China University of Technology, Guangzhou, China; h Department of Orthopaedics, Affiliated Hospital of Youjiang Medical University for Nationalities, Baise, China; i Menzies Institute for Medical Research, University of Tasmania, Hobart, Australia

**Keywords:** Intestinal microbiota, musculoskeletal system, influence, mechanism, intervention methods

## Abstract

The musculoskeletal system is important for balancing metabolic activity and maintaining health. Recent studies have shown that distortions in homeostasis of the intestinal microbiota are correlated with or may even contribute to abnormalities in musculoskeletal system function. Research has also shown that the intestinal flora and its secondary metabolites can impact the musculoskeletal system by regulating various phenomena, such as inflammation and immune and metabolic activities. Most of the existing literature supports that reasonable nutritional intervention helps to improve and maintain the homeostasis of intestinal microbiota, and may have a positive impact on musculoskeletal health. The purpose of organizing, summarizing and discussing the existing literature is to explore whether the intervention methods, including nutritional supplement and moderate exercise, can affect the muscle and bone health by regulating the microecology of the intestinal flora. More in-depth efficacy verification experiments will be helpful for clinical applications.

## Introduction

With the increasing severity of global aging, aging-related diseases, including sarcopenia, osteoporosis and osteoarthritis, have become increasingly prominent, affecting at least 7–27% of the world’s population.^1–3^ The incidence of sarcopenia ranges from 10 to 27%,^1^ the prevalence of osteoporosis among women over 50 years of age is no less than 20%,^2^ and the prevalence of osteoarthritis is approximately 7%.^3^ Despite tremendous efforts to control such diseases, there is less effective treatment available. At present, functional improvement and symptom control with the aim of improving the quality of life of patients remain the main goals in the management of musculoskeletal disorders. To shift from symptom management to individualized interventions targeting its pathogenesis, there is an urgent need to explore new pathogenic pathways and mechanisms, which could provide new therapeutic targets for musculoskeletal health-related diseases. In view of the subtle relationship between intestinal flora and its balance with body inflammation and metabolic function, emerging research has focused on the relationship between intestinal flora and its microecology and musculoskeletal health related diseases.

The gut microbial community is a complex but delicately balanced microecosystem composed of archaea, bacteria and eukaryotes, which exist as symbiotic entities in the gut.^4^ The intestinal flora is currently considered a new, complex organ composed of approximately 500 to 1000 species of microorganisms and 10^14^ bacteria, which is approximately 1.3 times the total number of human cells and more than 100 times the total number of human genes.^5,6^ It is affected by mode of birth, infant feeding, lifestyle, medication and host genetics. It is the largest microecosystem in the human body, and exhibits symbiosis with the host to maintain a dynamic equilibrium state of physiology.^7^ Although clear definitions of probiotics and detrimental microbes within the human gut microbiota are debatable, researchers tend to classify intestinal microbiota into probiotic, detrimental bacterial and conditioned bacterial strains.^8,9^

Intestinal microbiota is closely related to body health. Microorganisms in the intestinal tract participate in the onset and progression of various diseases and affect the physiological functions.^10^ Accumulating evidence shows that intestinal microorganisms and their metabolites exert a critical effect on the host immune system, nervous system, endocrine and metabolic functions. Thus, the intestinal flora is also called as “the second brain”.^11^ Recently, with the boom in molecular biology and high-throughput sequencing technology, significant progress has been made in research on the gut microbiota.^5^ Numerous studies have indicated that intestinal microecology plays an important role in maintaining gut homeostasis in the host and affects the progression of many diseases,^7,12^ such as neurodegenerative diseases,^13,14^ cardiovascular diseases^15^ and metabolic diseases.^16^ These biological mechanisms involve inflammation, anabolic metabolism, insulin sensitivity, nutrient bioavailability, toxin release and energy metabolism.^17,18^

Of note, the microbiota in the human intestine is not static, and their variety can change with age.^19^ Age-related changes in the gut microbiota are primarily manifested as intestinal ecological imbalance, a decline in bacterial diversity, an increase in opportunistic pathogens, and a reduction in the number of probiotics. It is not difficult to deduce that the gut microbiota participates in intricate mechanisms that lead to aging muscular dystrophy, such as sarcopenia.^20^

With thorough investigation, the impact of the intestinal microbiota on the musculoskeletal system has gradually come to light and has attracted the attention of researchers. Moreover, some researchers have proposed the concept of a “gut-muscle axis”^21^ and “gut-bone axis”.^22^ The composition and multiformity of the intestinal microbiota may be a determinant of metabolism and function in the musculoskeletal system. Dysbiosis and homeostasis of the gut microbiota can affect energy metabolism, immune and endocrine status, synthesis of secondary metabolites and host musculoskeletal homeostasis through the gut-muscle axis and gut-bone axis.^23,24^ This suggests that the intestinal microbiota is tightly associated with the functions of the musculoskeletal system and even mediates musculoskeletal diseases such as sarcopenia and osteoporosis (OP). Available data support microbial dysregulation (i.e., adverse changes in gut microbiota diversity) as a cause of disorders associated with systemic chronic low-grade inflammation, which is an important contributor to the pathogenesis of musculoskeletal disorders,^25^ while, the impact of intestinal microbiota on the musculoskeletal system, potential mechanisms and intervention methods have not been comprehensively and systematically reported. Based on the above considerations, we summarized and induced the interactions and intrinsic mechanisms between intestinal microbiota and the musculoskeletal system, such as muscle synthesis and breakdown, mitochondrial energy metabolism, oxidative stress injury, inflammation and osteoblast/osteoclast homeostasis. Following this, we summarized effective intervention measures to promote musculoskeletal health by regulating dysbiosis of the gut microbiota.

## Effects and mechanism of the intestinal microbiota on the musculoskeletal system

### Effect of “absence or presence of intestinal microbiota” on musculoskeletal system and its mechanism

#### Skeletal muscle

Experiments based on germ-free mice reveal the absence or presence of gut microbiota in vivo, which is crucial for the health of skeletal muscle by regulating material and energy metabolism pathways.^26^ The absence or presence of the gut microbiota in the body is critical for skeletal muscle health. A healthy intestinal microbiota can affect skeletal muscle function and metabolism through multiple pathways and mediators.^26^ Skeletal muscle mass was significantly lower in mice lacking gut microbiota, and the expression of myosin rejunction genes and skeletal muscle differentiation regulatory genes was significantly lower in germ-free (GF) mice than in normal germ-carrying mice, whereas the expression of muscle atrophy markers was significantly higher.^26,27^ Protein synthesis and degradation are important factors that affect skeletal muscle quality. The levels of insulin-like growth factor 1 (IGF-1), an important substance that promotes protein synthesis and enhances muscle function, decrease significantly in GF mice.^26,28^ Glucocorticoid-induced catabolism of branched amino acids is significantly increased in GF mice.^26,29^ Intestinal flora can also affect skeletal muscles by changing the patterns of energy metabolism. GF mice have lower glucose and insulin contents and significantly disrupted mitochondrial function, which affects the utilization of glucose by skeletal muscles, reduces the energy supply for muscle synthesis, and eventually affects the oxidative metabolic capacity of skeletal muscles.^26,30,31^ In addition, the level of choline decreased in GF mice, resulting in the impairment of neuromuscular junction transmission.^26,32,33^ Troponin is related to muscle fiber contractility and motor function in the skeletal muscles. The expression of the gene encoding troponin was reported to be markedly decreased in GF mouse skeletal muscle, indicating potential impairment of muscle fiber contractility.^26,34^ More importantly, after the intestinal microbiota was transplanted into GF mice, the results showed increased skeletal muscle mass, decreased muscle atrophy markers, improved muscle oxidative metabolism, and increased expression of neuromuscular junction assembly genes.^21,26^ This implies that intestinal microbiota plays a key role in maintaining normal skeletal muscle function.

#### Bone

The bone is continuously reconstructed through bone osteoblasts (OBs) and osteoclasts (OCLs). An imbalance in this process can lead to osteoporosis.^35^ Early studies have found that the intestinal microbiota is also a major adjuster of bone mass, and its impact on bone mass is mediated through its effect on the immune system, which in turn adjusts osteoclast genesis.^36^ Specifically, the loss of intestinal microbiota induces an abnormal increase in bone mass. Simultaneously, the number of osteoclast precursor cells and osteoclasts in the bone marrow of GF mice was reduced. Moreover, GF mice have decreased levels of gut-derived 5-hydroxytryptamine (5-HT) and inflammatory cytokines. Notably, colonization of GF mice with intestinal microbiota restored the lost bone mass. Of course, it cannot be excluded that other mechanisms may also involve in it.^37^ This result implies that the gut microbiota has a negative impact on maintaining bone mass. However, a recent study showed that colonization with gut microbiota from mice or humans does not cause bone loss in GF Swiss Webster and/or C57BL/6 mice.^38^ Furthermore, adult male GF BALB/c mice exhibit slower bone growth than normally raised mice.^39^ Long-term colonization of GF mice with fecal microbiota results in a surprising increase in femur length and improvement in trabecular bone microarchitecture.^40^ In addition, vitamin D has a great effect on improving intestinal calcium absorption to promote bone metabolism.^41^ It has been suggested that GF mice have defective vitamin D metabolism, while GF mice colonized by the microbiota indicated the restoration of 1, 25-dihydroxyvitamin D and calcium levels.^42^ Thus, the gut microbiota also has a beneficial effect on bone. These paradoxical results of gut microbiota on bone are partly due to differences in mouse strain, sex, age, content of colonization, or differences in antibiotic regimens, but the most likely reason may be the composition of the microbial community.^43^ Considering the critical role of intestinal microbiota in regulating bone health status, it is of great significance to probe the communication mechanism between the intestinal microbiota and host bone. This transfer of functional extracellular vesicles from specific microorganisms to bone tissue may be a critical mechanism for the transgene-mediated regulation of bone health.^43,44^

#### Joint

Accumulating evidence indicates that the gut microbiome plays a critical role in the pathology of osteoarthritis (OA).^45^ Earlier studies have shown that spontaneous T cell-dependent Rheumatoid Arthritis (RA) cannot be induced in IL-1αR^−/−^mice under GF conditions.^46^ In toll-like receptor 4 (TLR4)-deficient mice, RA manifestations were also significantly inhibited, and the gut microbiota was thought to act as an antigen or adjuvant to induce or assist RA production.^46,47^ The gut microbiota also has an impact on the development of traumatic OA. Studies have found that OA caused by medial meniscus instability surgery is significantly reduced in GF mice, suggesting that the intestinal microbiota promotes the development of OA.^48^ The gut microbiome has also been found to have a significant impact on the severity of injury-induced OA in a mouse model. After joint injury, GF mice had only mild OA symptoms, and the severity of the pathological manifestations was the lowest. Microbiome abundance in mice was positively correlated with inflammatory biomarker concentration, intestinal permeability and OA severity. This may be because, in the presence of joint injury, immune activation due to the gut microbiota (composed of more *Fusobacterium* and *Faecalis* and less *Ruminococcaceae*) may exacerbate the pathological process of OA.^45^

These animal experiments results indicate that the “presence or absence” of intestinal microbiota multiformity is indeed a critical factor about affecting and maintenance the musculoskeletal system. In the following section, we further elaborate on the effects and mechanisms of intestinal microbiota on the fitness of the musculoskeletal system from the perspectives of microbiota imbalance and probiotics along with their secondary metabolites.

### Effect of intestinal microbiota dysbiosis and its mechanism on musculoskeletal system

Intestinal microbiota dysbiosis refers to a state in which multiformity alternates within the intestinal microbiome. It mainly includes the bloom of pathobionts, reduction of normal flora and disappearance of the diversity of microbiota composition. Multiple factors, such as metabolic, autoimmune, inflammatory and neurodegenerative diseases, have been associated with intestinal dysbiosis.^49^ The influence of microbiota dysregulation on the musculoskeletal system has attracted increasing attention.

#### Skeletal muscle

Many factors are relevant to the pathology of sarcopenia, including aging, inflammation, mitochondrial damage, and/or insulin resistance. Interestingly, almost all of these pathological processes trigger dysregulation of the intestinal microbiota.^49^ In turn, microbiota disharmony also plays a crucial role in the decline in skeletal muscle mass and function. Since the gut microbiome is known to be responsible for the development and exacerbation of metabolic dysregulation phenotypes such as obesity or insulin resistance, skeletal muscle mass and function could partially regulate by gut microbiome.^23^

The major phyla of healthy gut microbiota are *Firmicutes*, *Bacteroidetes*, and *Actinobacteria*, while *Verrucomicrobia* and *Proteobacteria* are less extent. Similar gut dysbiosis was observed between the elder and the undernourished, both showing an increase in *Ruminococcus gnavus*, which promotes inflammation, and a decrease in *Roseburia intestinalis*, a butyrate producer.^50^ And *Proteobacteria phylum* seems to be more abundant in the gut microbiota of children with severe malnutrition.^51^ Rapid loss of muscle mass and changes in gut microbiota composition also occur in cachexia individuals. In cancer patients, the abundance of *Bifidobacterium*, *Lactobacillus*, and *Faecalibacterium* decreased, while *Enterobacteriaceae* and *Enterococcus* increased. Similar microbial colon patterns have been found in other patients with non-cancer cachexia who have chronic kidney, heart, or liver disease.^23,52^ The analysis of gut microbiota composition in the elderly population showed that microbiota composition changes with age. Individuals with high frailty scores show a significant reduction in relative abundance of *Lactobacilli*, *Bacteroides*/*Prevotella*, and *Faecalibacterium prausnitzii*, and increased *Enterobacteriaceae*, of which *Faecalibacterium prausnitzii* is considered an indicator of good gut health because it produces health-promoting short-chain fatty acids such as butyrate.^53–55^

The increase in circulating pro-inflammatory cytokines induced by gut dysbiosis can induce muscle atrophy via different mechanisms (insulin resistance, inflammation, and associated oxidative stress).^23,56^ Among them, muscle failure caused by inflammatory bowel disease (IBD)^57^ and aging^58^ are the two most typical examples of the gut microbiota dysbiosis. We use these two examples to specifically elaborate the intrinsic mechanism of sarcopenia induced by gut microbiota dysbiosis.

IBD is typically characterized by a dysregulation of the gut microbiota (primarily a decline in *Firmicutes* and *Bacteroidetes*, a relative heightened of *Entero bacteriaceae*) that disrupts the integrity of the intestinal barrier and then increases the pathogen penetration of intestinal tissues, thereby leading to lipopolysaccharide (LPS) translocation into the systemic circulation.^59^ Studies have demonstrated that gut microbiota dysregulation is often accompanied by intestinal barrier damage and an increase in intestinal barrier permeability, which eases the entry of endotoxins and other bacterial metabolites into the circulatory system, thus increasing the levels of LPS and other inflammatory factors and inducing inflammatory responses in the body.^58,60^ A characteristic manifestation of LPS-induced inflammation is severe muscle atrophy caused by increased proteolytic degradation and decreased protein synthesis.^61^ The diversity of intestinal microbiota may also lead to intestinal oxidative stress, intestinal mucosal inflammation and barrier dysfunction, thus causing immune dysfunction.^60^ Oxidative stress, caused by high levels of reactive oxygen species (ROS), can cause skeletal muscle contraction disorders, resulting in muscle weakness and fatigue.^62^ Therefore, inflammatory responses and oxidative stress caused by an imbalance in intestinal microbiota can negatively regulate muscle function. In a mouse model of colitis, skeletal muscle mass and muscle fiber cross-sectional area decreased and muscle protein content decreased in the quadriceps femoris and gastrocnemius muscles.^63^ At the same time, muscle dysfunction worsens and the muscle growth markers Insulin-like growth factor 1 receptor (IGF-1 R) and phospho-mammalian target of rapamycin (mTOR) are downregulated.^64^ Researchers believe that inflammation caused by dysregulation of the gut microbiota may be a triggering factor for skeletal muscle atrophy. The enhanced expression of muscle atrophy F-box (atrogin-1) and muscle ring finger protein 1 (Murf-1), which are related to the breakdown of myofibrils, mediates the accelerated breakdown of myofibrils.^65^ Clinical studies have also found that muscle injury is a common pathological feature of chronic gastrointestinal diseases such as IBD. Sarcopenia occurs in 42% of IBD patients.^66^ The intestinal microbiota stimulates mucosal immune cells to promote pro-inflammatory cytokines (interleukin 6 (IL-6), interleukin 10 (IL-10), tumor necrosis factor alpha (TNF-α), etc.), produce the general state of chronic low-grade inflammation, activate oxidative stress injury, and further influence insulin sensitivity, amino acid biosynthesis, mitochondria, biological generation, the synthesis and catabolism of muscle and the increase muscle attenuation-related molecular pathways. This can lead to musculoskeletal damage and weakness.^67–69^

Similarly, the multifarious composition of the intestinal microbiota in frail or motile elderly also shows varying levels of dysregulation, with reduced species richness and an imbalance between opportunistic pathogens and anti-inflammatory flora.^53,70^ Dysregulation of the gut microbiota may be related to the complicated mechanisms of muscular atrophy.^58,71^ Specifically, the intestinal microbiota diversity decreases due to aging. Simultaneously, the ability to regulate the gut environment also decreases, the gut barrier function weakens, and the gut mucosal permeability increases, which collectively leads to increased absorption of bacterial products, including LPS, and activation of the inflammatory response in the body.^72^ The gut microbiome associated with aging promotes inflammation so that levels of circulating inflammatory mediators increase, and reversing these age-related microbiome changes is a potential strategy to reduce age-related inflammation and accompanying morbidity.^72^ LPS promotes inflammatory signaling, induces inflammation and insulin resistance in skeletal muscles, thereby contributing to the process of metabolic syndrome, and then induces skeletal muscle aging.^73–75^ Studies showed that LPS-related cytokines decisively affect the ability to balance (i.e., synthesis and breakdown) proteins, and that increased cytokines with aging may induce muscle mass reduction.^76,77^ An enhanced inflammatory response can aggravate skeletal muscle mass loss. For example, in patients with sarcopenia, increased levels of pro-inflammatory factors such as IL-6 and TNF-α, among others, have been reported to induce a decrease in muscle mass.^78^ Anti-TNF treatment can reverse age-related changes in the microbiota and therefore has potential anti-muscle aging effects.^72^ The study also found that changes in the diversity and composition of the gut microbiota are associated with the disturbance of immune homeostasis during aging. The small intestines of young germ-free mice transplanted with the gut microbiota of older mice identified immune-related differential genetic signatures, including reduced antigen presentation and altered cytokine and chemokine production. These genes may play a potential role as markers of immune disorders during the aging of the gut microbiome.^79^

Changes in the composition of the gut microbiota ecosystem due to various of the aforementioned causes (inflammation, malnutrition, cachexia, aging, etc.) may lead to intestinal leakage and the release of bacterial endotoxins (e.g., LPS) into the peripheral blood. LPS can trigger macrophages to produce inflammatory cytokines and ROS through TLR4 receptor. In skeletal muscle, TNF-α activates the expression of genes involved in the nuclear factor-κb (NF-κB) pathway, thereby reducing muscle cell differentiation and proliferation by inhibiting myogenin and myoD. IL-6 and IκB kinase can inhibit insulin receptor substrate 1, which is associated with the induction of insulin resistance, limiting the activation of muscle targets of rapamycin complex 1 (mTORC1), thereby limiting protein synthesis in muscle cells. In addition, the inhibitory effect on forkhead box O can no longer be exerted due to the inhibition of protein kinase B, which leads to increased expression of ubiquitin E3 ligases, Atrogin-1 and MuRF1, promoting muscle growth proteolysis. Similarly, autophagy activating kinase 1, which is not inhibited by mTORC1, further induced autophagy in skeletal muscle cells. When these regulatory mechanisms induced by gut dysbiosis are activated, an imbalance between protein breakdown/synthesis occurs and ultimately leads to muscle atrophy.^23^

Other mechanisms include that the quorum-sensing peptide iAM373 produced by *Enterobacter faecalis* during bacterial dysregulation downregulated most skeletal muscle development and differentiation genes, reduced the metabolic activity of myoblast cells, and upregulated the proteasome degradation pathway in muscle ducts, which is a new inducer of sarcopenia.^80^ Cohort research has suggested that sarcopenia is also correlated with a decrease in short-chain fatty acid (SCFAs)-producing gut microbiota, resulting in an imbalance in gut flora.^81,82^ Dysregulation of the intestinal flora may lead to skeletal muscle atrophy through upregulation of the primary bile acid-farnesoid X receptor pathway.^83^

#### Bone

As the critical role of intestinal microbiota in the bone metabolic steady state is better understood, there is growing interest in the important role of gut microbiota in regulating bone health^84^. Researchers have shown a strong relationship between intestinal microbiota dysbiosis and bones, which suggests that the intestinal microbiota regulates bone metabolism^24,85^. Varying degrees of evidence, both from animal models and human studies, support the close connection between gut microbiota dysregulation and bone health or disease^86^.

It was found that the damage of intestinal microbiota and poor microbial diversity in TLR5-deficient mice induce changes in whole bone strength;^87^ In addition, the damage of intestinal flora caused by long-term use of antimicrobials (mainly characterized by the obvious decline of *Bacteroides* and the significant upregulation of *Proteobacteria*) lead to the damage of bone performance in mice, especially in the reduction of total bone strength.^88^ Ovariectomy induces dysregulation of gut microbiota and leads to bone mass loss in mice, which is mediated by microbe-dependent T lymphocytes (such as T helper cell 17 (Th17) cells).^89^

Changes in the intestinal microbiota have been observed in patients with skeletal disorders. Existing cross-sectional studies have shown an association between intestinal microbiota and the accumulation of bone mineral density.^90^ Recently, a genome-wide study identified the gut bacterial taxa *Clostridiales* and *Lachnospiraceae* related to bone mass variability, revealing a causal link between microbiota and bone development.^91^ The study found that patients with bacterial overgrowth had lower bone mass and higher rates of bone loss than healthy people.^92^ Aggravated bone loss is a common complication of IBD, and dysregulation of the gut microbiota is also an important manifestation of IBD.^93,94^

Clinical studies have shown that the composition and abundance of the intestinal microbiota in individuals with low bone mineral density are significantly decreased. Bacterial groups, including *Roseburia*, *Bifidobacterium* and *Lactobacillus* were positively correlated with bone mineral density. However, in people with low bone mineral density, the microbiota associated with LPS synthesis is more abundant.^95^ The *Firmicutes*/*Bacteroidetes* ratio usually represents the unbalance of intestinal flora in different degrees or states. The study found that the *Firmicutes/Bacteroidetes* ratio was negatively associated with bone mass, and the abundance of *Actinobacteria* and *Bifidobacteriaceae* was positively correlated with bone volume. Analysis of gut microbiota in OP patients using 16s RNA sequencing showed that gut microbiota diversity estimates were negatively correlated with bone mineral density.^96,97^ In patients with OP, an increase in the abundance of representative bacterial genera was observed, such as *Actinomyces*, *Clostridium Cluster XlVa*, *Eggerthella*, *Blautia*, *Lactobacillus*, *Ruminococcaceae* and *Parabacteroides*.^98,99^ Other studies have found that the diversity of intestinal microbiota in OP patients, particularly the quantity of *Dialister* and *Faecalibacterium* increased significantly.^100^

The microbiota has a necessary effect on nutrient transport and absorption, which are required for bone health, such as calcium and vitamin D.^101^ Dysregulation of gut microbiota may impair the transport of nutrients and calcium through the gut into the circulatory system. The decrease in intestinal absorption capacity of 1, 25(OH) _2_-D3 and calcium increases with age, which is closely related to the dysregulation of intestinal microbiota.^102^ This is one of the primary reasons that microbiota dysbiosis affects bone health.

In addition, the immune and inflammatory systems are thought to intervene between the gut microbiota and bone metabolism. The change in biodiversity and colonization of opportunistic pathogens leads to an increase in bacterial endotoxins, such as LPS,^103,104^ which is related to an increase in the intestinal inflammatory response, and the increase in inflammation is related to the activation of osteoclasts. Intestinal flora dysregulation mediates inflammation, especially Interleukin 1 (IL-1), TNF-α and IL-6, which play a key role in osteoclast activation as long as OP.^105,106^ The proliferation of intestinal microbiome-dependent Th17 cells and TNF-α-producing T cells produces a large number of pro-inflammatory cytokines (IL-17 and TNF-α), receptor activator of NF-κB ligand (RANKL),^107^ and decreases the secretion of RANKL antagonists (RANKL induces osteoclast function while IL-17 reduces bone formation; TNF-α enhances the activity of RANKL and induces Th17 cell proliferation and activation), which may be the underlying immune regulatory mechanism in the process of OP.^108^ Th17 cells are a population of osteoclasts that produces IL-17 and secretes TNF-α and RANKL. Interestingly, newly generated osteoclasts induced the generation of Treg cells.^109^ It suppresses immune responses, induces and maintains immune tolerance, reduces inflammation through several pathways, and yields immunosuppressive cytokines, such as TGF-β and IL-10.^110^ The subtle and complex relationship between Treg and Th17 cells can influence bone health. Crucially, gut bacteria are key to controlling this balance.^86^

5-HT is classified into two types according to where it is synthesized: brain-derived and gut-derived 5-HT. Interestingly, these two 5-HT have different functions: gut-derived serotonin has a negative impact on bone formation, whereas brain-derived serotonin has an opposite influence.^111^ In recent years, studies have found that gut flora not only induces cytokines to adjust bone metabolism, but also downregulates gut-borne serotonin levels by reducing the serotonin biosynthesis enzyme and increasing serotonin transporters, thereby regulating bone metabolism.^37^

IGF-1 also plays a crucial role in the regulation of bone formation and growth. Gut microbiota may regulate overall bone quality by altering IGF-1 levels. For example, colonization of adult GF mice by conventionally specific intestinal microbiota can increase circulating IGF-1 and increase the formation and resorption of bone.^40,112^ Reduced gut microbiota diversity may also lead to a decrease in circulating estrogen, which in turn affects normal bone calcium deposition because the intestinal microbiota adjusts estrogen through β-glucosidase secretion, which breaks down estrogen into its active form.^113^

According to the above research results, it is suggested that, similar to muscle health, bone health-related intestinal flora disorder is mainly manifested by the dominant intestinal flora, leading to excessive LPS production or causes more inflammatory reactions. Mechanisms mainly involve the subtly controlled osteogenic/osteoclastic balance of cytokines and Treg and Th17 cells. Difference from muscle health, the effect of gut dysbiosis on the effective absorption of calcium ions and the regulatory effects of gut-derived 5-HT, IGF-1, and estrogen are also highlighted here. Whether these mechanisms also have a role in muscle health may be a meaningful direction of exploration.

#### Joint

Dysbiosis of intestinal flora is involved in the pathogenesis of OA. Abnormalities in intestinal microbiota can seriously impair joint function.^25,45^

Fecal microbiota transplants from patients with impaired metabolism accelerate OA in mice, and dysregulation of the gut microbiota (e.g., abundance changes of *Ruminococcus, Faecalis* and *Fusobacterium*) may aggravate the severity of cartilage and subchondral bone pathology in OA mice.^45^ In mice, variations in the intestinal flora induced by ampicillin and neomycin reduced serum LPS levels and suppressed inflammatory responses. This leads to improvement in OA after joint injury.^114^ Chronic antibiotics also attenuate cartilage injury by altering the multiformity of the gut flora in mice with a spontaneous metabolic syndrome phenotype.^88^

Population studies have found that the abundance of *Lentisphaerae* was inversely associated with the OA prevalent.^115^ And the abundance of intestinal streptococci is correlated with intensive knee pain and inflammation.^116^ But existing studies only weakly support the relationship between the gut microbiome and OA, particularly the association between certain groups or microbial products and inflammatory status and OA symptom severity (including knee pain), and there is a lack of high-quality studies on the potential role of the gut microbiome in OA-related pain.^117^

Studies have suggested that the prevalence of OA is possibly due to the specific changes in gut microbiota composition observed in the gut ecosystem of the elderly, in particular an increase in opportunistic pro-inflammatory bacteria and a significant decline in symbiotic bacteria with anti-inflammatory properties.^118^ Alterations in intestinal permeability may explain the influence of intestinal microbiota on the emergence and persistence of inflammatory diseases. Once the mucosal barrier is disturbed, components of the exogenous lumen invade the gut environment and mucosal inflammation and immune activation ensue.^119^ Gut bacteria can bias the immune system toward a pro-inflammatory state, which can eventually lead to joint inflammation.^120^ An increase in intestinal barrier permeability due to an imbalance in the intestinal microbiota was positively correlated with OA severity in mice.^45^ Intestinal microbiota may mediate OA through the translocation of microbial molecules into the circulatory system. LPS is produced by gut microbiota and migrates to the circulatory system, contributing to low-grade inflammation. Metabolic endotoxemia, which involves the interaction between gut-derived LPS and TLR4, has recently been recognized as a common source of low-grade inflammation, including OA.^121^ The study also confirmed that serum LPS levels were positively correlated with the number of activated macrophages in the knee synovium and the severity of arthritis.^122^ The intestinal microbiota affects Tregs in the colon, suggesting that intestinal microbiota also modulates intestinal immune tolerance.^123^ These interactions also modulate inflammation and autoimmune diseases such as OA.^124^

In recent years, increasing evidence has reported that dysregulation of the intestinal microbiota is strongly associated with the occurrence and development of RA. Intestinal microbiota dysregulation has also been observed in RA in human and animal studies.^120,125^ Significant changes in gut microbial communities were observed in RA model mice induced by collagen emulsion, mainly characterized by a decline in *Bacteroidetes* and an increase in *Firmicutes* and *Proteobacteria*. The model also resulted in an imbalance of 14 intestinal bacteria and considerable interference with metabolites involved in tryptophan, fatty acids and secondary bile acids.^125^

In patients with RA, various *lactobacillus* and *Prevotella* genera have been found to be more abundant; therefore, increased quantities of *Prevotella* and imbalance of gut flora have been suggested as latent resources for the development of RA.^126^ Dysregulation of gut microbiota in RA patients was correlated with the severity of RA; *Euryarchaeota* was directly correlated with the severity of RA, becoming an independent risk factor for the pathogenesis of RA.^127^ There was a strong correlation between disease and the presence of *Prevotella* and the loss of *Bacteroides* in the fecal microbiota of untreated patients with new-onset RA. Bacterial rRNA was isolated from the synovial tissue of patients with RA.^120^ The quantity and multiformity of *Lactic acid bacteria* in the gut microbiota of RA patients were significantly increased.^126,127^ This is consistent with the data that reported an increase in *Lactobacilli* in mice with collagen-induced arthritis.^128^ Paradoxically, *Lactobacillus acidophilus* and *casei* seem to be conducive to the improvement of RA, indicating that different *Lactobacillus* subsets may have different effects on the pathogenesis of RA.^129^

Intestinal opportunistic pathogenic bacteria, such as *Prevotella*, may participate in the formation of a pro-inflammatory immune state by enhancing the apoptotic mechanism and disrupting gut barrier integrity, thereby inducing the occurrence and maintenance of arthropathy inflammation.^130^ In addition, Th17 cells promote osteoclast differentiation by producing a series of inflammatory factors, which are thought to be responsible for the osteodestructive phase of RA.^131^ The reduction of Bacteroides, also found in RA mice, may promote a localized inflammatory environment by reducing Treg cell differentiation.^125^

The occurrence of OA may be due to specific changes in the composition of intestinal microbiota, especially the increase of opportunistic pro-inflammatory bacteria and the significant decrease of commensal bacteria with anti-inflammatory properties, the increase of intestinal permeability, and then LPS causes inflammatory and immune responses, inducing the activation of cascade signaling pathways, leading to joint lesions and even pain, but the relevant evidence is limited. However, the study of RA related to immune inflammation is relatively deeper, and the diversity of intestinal flora is richer, but it also leads to the more complexity of research results and biological mechanisms, which needs further exploration of the rules and truth.

### Effect of probiotics along with their secondary metabolites and its mechanism on musculoskeletal system

The concept of ‘probiotics’ was first coined in 1974, that is, ‘living microbes that have health benefits. Gut probiotics are beneficial for host health and body function.^132^ Here, we summarize the effects and mechanisms of probiotics and their secondary metabolites on the musculoskeletal system.

#### Skeletal muscle

The gut is linked to skeletal muscle due to the activity of the gut microbiome and regulates muscle function by regulating systemic/tissue inflammation, insulin sensitivity, etc. Probiotics combat loss of muscle mass and function by improving the diversity of the gut microbiota.^23^ In mouse cancer models, *Lactobacillus reuteri* is able to inhibit the development of cachexia and is implicated in the preservation of muscle mass.^133^ *Lactobacillus plantarum* HY7715 ameliorates sarcopenia by improving skeletal muscle mass and function in aged Balb/c mice.^134^ *Lactobacillus plantarum* TWK10 supplementation improves exercise performance and increases muscle mass in mice.^135^ *Lactobacillus paracasei* PS23 decelerated age-related muscle loss by ensuring mitochondrial function in SAMP8 mice.^136^ Restoring specific lactobacilli levels decreases inflammation and muscle atrophy markers in an acute leukemia mouse model^137^. Other experiments have also found that probiotics LC122, BL986 and LPPS23 can improve intestinal microflora imbalance and prevent age-related loss of muscle mass and strength in elderly mice.^136,138^ In a word, recent reports suggest that there are at least seven probiotics (*Lactobacillus casei LC122* (LC122), *Saccharomyces boulardii* (SB), *Lactobacillus paracasei PS23* (LPPS23), *Bifidobacterium longum BL986* (BL986), *Lactobacillus plantarum TWK10* (LP10), *Lactobacillus salivarius SA-03* (SA-03) and *Bifidobacterium longum OLP-01* (OLP-01)) is beneficial to skeletal muscle mass and strength in mice.^139^ Among them, the most widely used strains are *Lactobacillus* and *Bifidobacterium*, which can improve muscle mass, strength and endurance loss.^23,132,139^ Although recent studies have shown that probiotics that can limit sarcopenia or promote health performance in rodents, In anabolic situations, the effects of probiotics may target different signaling or metabolic pathways and tissues, such as lower inflammation and stress, maintenance of muscle protein synthesis, and improved muscle strength. However, the data obtained after supplementation of probiotics in the catabolic animal model showed that the effect on muscle mass and function was almost small, and only a certain anti-inflammatory effect was observed.^23^

In humans, it has been found that ingesting specific probiotics can alter the gut microbiota in favor of increased skeletal muscle mass.^140^ For example, a six-week intake of TWK10 can improve endurance performance on running tests in young adults.^141^ Studies of supplementation with *Lactobacillus plantarum* have shown that probiotics improve exercise performance, endurance, as well as body composition, reducing fat mass and increasing muscle mass.^142^ However, it has also been reported that, to a certain extent, probiotic supplementation in the elderly population can lead to beneficial changes in the gut microbiota, reducing pathogens and improving constipation, but the impact on host health is relatively small.^23^ Fermented milk containing *Lactobacillus* and *Bifidobacteria* strains, as well as *Lactobacillus casei*, has also been reported to have no effect on muscle condition in the elderly.^143^ These findings suggest that the mechanism of action of probiotics is complex and requires further investigation. Therefore, a recent meta-analysis suggested that probiotic supplementation enhanced muscle mass and strength, however, no beneficial effects were observed in terms of total lean body mass. The study suggests that it is important to explore differences in physiological mechanisms among different aging populations and to explore supplementing with appropriate probiotic strains for optimal muscle mass and strength.^144^ From the above studies, it is not difficult to find that, the scarcity of studies, variability of populations, and low reproducibility make it difficult to find specific probiotic strains for optimizing muscle mass and function, and further studies in more defined populations are needed to design personalized probiotic interventions.^23^

Probiotics reduce the ratio of *Firmicutes* to *Bacteroidetes*, which improves muscle mass, endurance and strength in mice.^145^ The addition of probiotics LP10 and CP2998 can play a positive role in muscle by inhibiting glucocorticoid receptor activation and improving glucose.^135^ Supplementation with LC122 and BL986 decreased the expression of the inflammatory cytokines TNF-α, IL-6 and IL-1β and improved muscle protein synthesis.^136,138^ Oral administration of *B. breve* and *Lactobacillus rhamnosus* significantly reduced the levels of the pro-inflammatory cytokines IL-2, IL-4, IL-6 and TNF-α in mice, thereby suppressing inflammatory responses.^146^ LPPS23 also alleviated muscle inflammation by increasing IL-10 levels.^136^ The expression of antioxidant stress factors, such as superoxide dismutase and glutathione peroxidase, was higher in the muscle of aged mice administered LPPS23.^136^ Supplements containing LP10 and *Lactobacillus reuteri* were also found to improve muscle mass in mice, which was associated with reduced inflammation and muscle atrophy marker expression.^135,137,147^ Long-term probiotics (such as *L. reuteri*) boost the leucine absorption ratio from whey protein to enhance protein absorption, improve the protein utilization rate and eventually promote muscle synthesis and increase muscle mass.^148^

Therefore, regulating the composition of the intestinal flora is one of the mechanisms of action of probiotics, and the activity and diversity of the microbial community may be a determinant of healthy muscle under various pathological conditions.^23^ The beneficial effects of probiotics also occur through various mechanisms, including the induction of immune regulation, resistance to physiological stress, inhibition of pathogens and improvement of the barrier function of intestinal epithelial cells.^132^ The potential mechanisms by which probiotics regulate muscle mainly include energy metabolism of glucose, lipids and proteins; inflammatory levels; mitochondrial function and neuromuscular connectivity; and molecular pathways of muscle synthesis and catabolism metabolites.^139^ Gut probiotics break down carbohydrates, lipids and proteins to provide energy to their hosts. They have also been shown to limit insulin resistance, regulate metabolic pathways, or inhibit oxidative stress and inflammation. In addition, probiotic-derived SCFAs and bile acids are involved in regulating host metabolism.^7,149,150^

The intestinal microbiota can also decompose nutrients into secondary metabolites through distinct metabolic pathways, regulate intestinal immunity and metabolic homeostasis, and maintain symbiotic and parasitic relationships between the host and the intestinal microbiota.^151^ The primary effects and mechanisms of action of these secondary metabolites on the skeletal muscle metabolism are discussed below.

SCFAs are metabolic products produced in response to dietary fiber fermentation by intestinal flora and primarily include butyrate, propionate and acetate.^152^ SCFAs play an important role in glucose and lipid homeostasis, adjustment of inflammation, and connections between the gut and other distant tissues.^23^ SCFAs play an important role in regulating intestinal environmental homeostasis, improving glucose metabolism, promoting calcium and phosphorus absorption, and alleviating oxidative stress and inflammatory responses, which are of great significance for regulating skeletal muscle functions.^153,154^ Skeletal muscle injury was partially reversed upon treatment with SCFAs in GF mice (SCFAs decreased atrogin-1 expression and increased MyoD and muscle mass and function), suggesting that SCFAs produced by the gut flora play a critical role in regulating skeletal muscle function.^26,155^ In a study of female C57BL/6 mice, atrophy of the hind limb muscle was completely or partially ameliorated in response to butyrate treatment, whereas the mass of the hind limb muscle was significantly lower in aging C57BL/6 female mice fed a regular diet.^156^ Butyrate was shown to not only preserve muscle mass, but also improve glucose tolerance in mice, whereas it exerted no significant effect on insulin tolerance. In addition, butyrate increases the levels of the mitochondrial protein porin and mitochondrial transcription factor A and significantly improves mitochondrial function in skeletal muscle cells.^156,157^ Moreover, butyrate treatment reduces oxidative stress expression and markers of apoptosis in mice and alters the activity of antioxidant enzymes, thus preventing skeletal muscle damage caused by oxidative stress.^156^ Butyrate is an important SCFA and histone deacetylase inhibitor that regulates age-related muscle loss. Butyrate has been shown to promote muscle synthesis by inhibiting histone deacetylase expression and improving muscle mass and cross-sectional area in aged mice.^156,158^ The addition of acetate can promote glucose absorption and glycogen generation in rabbit skeletal muscle and reduce intramuscular lipid production by increasing fatty acid mobilization and oxidation.^159^ Additionally, SCFAs induce IGF-1 production, which promotes muscle anabolism.^40^ These metabolic impacts of SCFAs may act directly on skeletal muscle or may be produced indirectly by stimulating glucagon-like peptide 1 (GLP-1) secretion. Other indirect effects of SCFAs on muscles include accelerated blood flow effects.^160^

Tryptophan is an indispensable aromatic amino acid in humans. Metabolites rich in indole and indole derivatives produced during the decomposition of tryptophan by the intestinal microbiota play a critical role in maintaining homeostasis in the intestinal environment and the diversity of intestinal flora.^161^ Indole metabolites derived from the intestinal microbiota can enhance body IL-10 levels, and IL-10 has an anti-inflammatory effect in regulating the inflammatory state of the host.^162^ Evidence from other studies suggests that the tryptophan metabolite indole acrylic acid promotes intestinal barrier function and suppresses the inflammatory response by downregulating inflammation- and oxidative stress-related gene expression. The anti-inflammatory effects of tryptophan metabolites are particularly important when the body is in a chronic inflammatory state and muscle protein synthesis is limited.^163^

Bile acids are small metabolic molecules that are produced in the liver and secreted into the intestine, and are involved in dietary lipid absorption. Intestinal flora can change the structure, bioavailability and bioactivity of bile acids, thus affecting bile acid metabolism and host metabolic homeostasis.^164^ Intestinal anaerobes have been shown to transform primary bile acids into secondary bile acids.^165^ The *Lachnospiraceae* family can also produce SCFAs and switch from primary bile acids to secondary bile acids.^166^ Intestinal flora may be involved in bile acid metabolism and the FXR-FGF19 signaling pathway (Evidence suggests that FGF19 activation can inhibit the protein expression of muscle atrophy markers, enhance the molecules associated with myogenic differentiation, and regulate skeletal muscle protein balance,^83^ which in turn affects skeletal muscle metabolism.^167,168^ Secondary bile acids can also enhance muscle function by contributing to lipid and glucose metabolism.^169^

Vitamins are essential micronutrients for humans and the body cannot synthesize most of them. Experimental evidence has confirmed that intestinal probiotics promote the synthesis of vitamins in the body, which have a significant impact on skeletal muscle function. For example, *Bifidobacterium* and *Lactobacillus* have been shown to synthesize vitamin B groups (including folate, riboflavin and vitamin B_12_).^170^ B-group vitamins are water-soluble vitamins directly involved in energy metabolism, and their deficiency has been reported to cause myocardial damage and heart failure.^171^ Vitamin B_12_ deficiency increases homocysteine levels and causes muscle damage.^172^

#### Bone

Previous studies have shown that probiotics have been found to benefit intestinal homeostasis and play a key role in the prevention and treatment of bone mass loss.^173^ The upregulation of *Lactobacillus* in the gastrointestinal tract may be crucial for promoting the symbiosis of intestinal microbiota and intestinal barrier function, as *Lactobacillus* can produce effective bactericides and organic acids, which can inhibit pathogenic *Escherichia coli*.^24^ The increased abundance of *Lactobacillus* and *Lactococcus* in the gut flora can effectively improve gut microbiota composition, thus promoting bone growth.^174^ Supplements with *L. reuteri* can significantly improve any imbalance in intestinal microflora and enhance intestinal barrier function to prevent bone loss.^175^ In a randomized controlled trial, postmenopausal women aged 75–80 years with low bone mass treated with a daily dose of *L. reuteri*, the proportion of bone mineral density loss after 12 months was obviously lower than that in the control group, suggesting that probiotics reduced bone mass loss.^176^ Supplementation with *B. vulgatus* can also reduce LPS concentration to suppress the inflammatory response and prevent bone loss.^177^ *L. acidophilus*^178^ and *Bacillus clausii*^179^ can maintain bone homeostasis by balancing the levels of inflammatory cytokines.^24^ *B. longum* has also been shown to prevent and treat osteoporosis, improve bone loss by enhancing osteoblast activity, and inhibiting osteoclast formation.^180^ Additionally, probiotics can improve bone health by regulating mineral absorption. For example, *B. longum* can enhance bone density by improving the absorption of minerals, such as calcium, phosphate and magnesium.^181^ *L. reuteri* has been confirmed to alleviate bone mass loss by inhibiting osteoclasts.^182^

SCFAs also participate in bone metabolism and affect bone formation and resorption.^24^ The protective effect of SCFAs on bone mass is related to the suppression of bone resorption. In terms of the underlying mechanism, butyrate and propionate induce the osteoclasts metabolic reprogramming, enhance glycolysis, downregulate critical osteoclast genes, and significantly reduce the number of osteoclasts, thereby inhibiting bone resorption. Therefore, SCFAs are effective adjusters of osteoclast metabolism and bone homeostasis, and play important roles in promoting bone formation.^183^ In a study in mice, butyrate was found to promote bone anabolism and increase bone mass by mobilizing the Wnt signaling pathway in osteoblasts.^110^ Moreover, butyrate can protect osteoblast precursor cells from damage induced by hydrogen peroxide and promote mineralization and differentiation of osteoblasts. It primarily maintains the balance of bone metabolism by enhancing the activity of cellular antioxidant enzymes, promoting ATP production, and reducing ROS levels.^184^ Additionally, SCFAs induce IGF-1 production. IGF-1, in addition to promoting skeletal muscle function, and plays a crucial role in bone metabolism. IGF-1 participates in bone formation and absorption and regulates the balance of bone metabolism.^40^ Thus, SCFAs play a critical role in maintaining homeostasis during bone metabolism.

Tryptophan metabolites are closely related to bone metabolism. Kynurenine, a tryptophan metabolite, is the first stable metabolite formed after the enzymatic degradation of tryptophan.^185^ The key function of kynurenine in bone metabolism appears to be the acceleration of bone loss and the mediation of adverse effects on the bone. Kynurenine content increases during aging, and its adverse effect on bone may be due to its effect on osteoclast activation, resulting in increased bone fragility and an imbalance in bone remodeling.^186^ Other studies have shown that increased kynurenine levels accelerate bone aging by impairing osteoblast differentiation and increasing osteoclast resorption.^187^

Low vitamin B_12_ levels can also inhibit osteoblast activity, thus increasing osteoporosis risk and even inducing fracture.^188^ Probiotics, such as *lactic acid bacteria* and *Bifidobacteria* play a critical role in promoting the formation of riboflavin and folic acid.^170,189^ Riboflavin and folic acid are important for promoting vitamin synthesis and regulating the inflammatory response. Studies have shown that riboflavin plays an anti-inflammatory role to an extent, and its intake inhibits TNF-α release in rat leukocytes.^190^ Riboflavin also regulates oxidative stress as a cofactor for antioxidant enzymes.^191^ Folic acid has been implicated in regulating insulin resistance and suppressing the pro-inflammatory cytokines IL-6, IL-8 and TNF-α.^192^ It is not difficult to find that the functions of vitamin B group such as anti-inflammatory, antioxidant and participating in energy metabolism are indispensable in the maintenance of skeletal health.^86^ The absorption of calcium and vitamin D is particularly crucial for healthy bone maintenance, probiotics reduce intestinal PH and improve calcium absorption to enhance bone function.^193^

Secondary bile acids also modulate bone homeostasis by regulating signal transduction between osteoblasts and osteoclasts.^194^ In addition, secondary bile acids induce the production of GLP-1, which regulates glucose homeostasis and stimulates osteoblast differentiation^24,195^ and can further enhance the functions of the skeletal system.

#### Joint

In a rat model of OA, the probiotic complex attenuated the development of osteoarthritis by inhibiting pro-inflammatory cytokines and cartilage destruction.^196^ In a human trial, 537 OA patients were randomized to either *L. casei* or a placebo, and systemic inflammation was significantly lowered after 6 months in the experimental group compared to that in the control group.^197^ Oral intake of *Streptococcus thermophilus* improves OA degeneration.^198^ Oral administration of *Clostridium butyricum* can effectively preserve knee cartilage and synovium in OA rats, significantly reduce the amount of fibrous tissue, and significantly reduce the serum concentrations of various inflammatory and metabolic markers of bone and cartilage.^199^ *Lactobacillus casei* alleviates joint inflammatory damage by downregulating pro-inflammatory cytokines.^200^

Probiotics have recently been added to the list of treatment agents for joint inflammation, as *L. casei* was shown to inhibit joint swelling, reduce RA, and prevent bone destruction in rats with joint inflammation.^201^ *Lactobacillus acidophilus* and *Lactobacillus casei* are also commonly used as relievers to manage RA.^129^ It has also been suggested that the availability of probiotic supplements to relieve RA-related outcomes is still weak, and the impact of probiotics on RA appears to be flora specific.^202^

SCFAs can reduce arthritis severity by regulating IL-10 secretion from Treg cells.^203^ Butyric acid produced by *Lactobacillus* inhibits OA by controlling chondrocyte autophagy and inflammatory cell death.^204^ Probiotic-derived butyrate has been shown to inhibit mouse arthritis by influencing T and B cell progression.^205^

**Figure 1.**
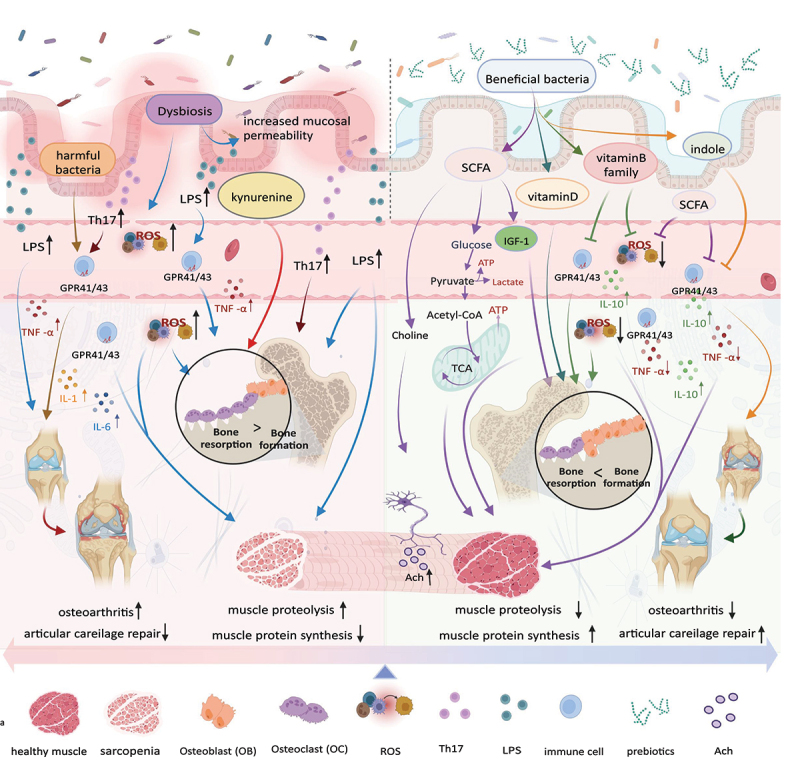
Intestinal microbiota and the metabolites they produce play a crucial role in the musculoskeletal system. Probiotics promote short-chain fatty acids (SCFAs) production, which enhance the function of the musculoskeletal system by improving energy metabolism, promoting IGF-1 production, and inhibiting inflammation and oxidative stress. Vitamin B promotes musculoskeletal development by reducing inflammation and oxidative stress, whereas vitamin D enhances bone function by promoting bone formation. Indoles promote musculoskeletal development by reducing inflammation. Prebiotics enhance the function of probiotics function. However, in addition to the positive effects, imbalances in the intestinal microbiota can trigger a series of negative effects. First, an imbalance in intestinal permeability promotes the increase in the ROS and LPS levels in circulation and causes oxidative stress and inflammation, which damages the musculoskeletal systems and joints. Second, harmful bacteria can activate the inflammatory response and suppress musculoskeletal function. Kynurenine can enhance osteoclast function, increase bone resorption, and promote bone loss. Created with BioRender.com.

As for probiotics and their secondary metabolites, current research results are limited. However, its anti-inflammatory and immunomodulatory properties warrant further investigation to determine its roles and biological mechanisms in the musculoskeletal system. In summary, we present the primary mechanisms discussed above in concise form in Figure 1. We also summarize the findings of related studies published in recent years on the influence of common intestinal microbiota on the musculoskeletal system, as shown in Table 1.

**Table 1.** The influence of common intestinal microbiota on the musculoskeletal system.

	Involving intestinal microbiota	Subject	Sample size	Means of intervention	The experimental results	Year	Ref.
Skeletal muscle	*Lactobacillus reuteri*, *Lactobacillus gasseri*	BALB/c mice (5-week-old)	31	Added to drinking water (2 × 10 ^8^ CFU/mL)	Restored *Lactobacillus* levels and suppressed the expression of proinflammatory factors and muscle wasting markers	2012	^137^
	*Lactobacillus plantarum*	ICR mice (6-week-old)	24	Oral (2.05 × 10^8^ CFU/kg and 1.03 × 10^9^ CFU/kg)	Increased the relative muscle mass and the number of type I fibers in the gastrocnemius muscle and improved muscle strength	2016	^135^
	*Lactobacillus reuteri*	CD2F1 mice (8-week-old), BALB/c mice (6-week-old)	8	Added to drinking water (2 × 10^8^ CFU/mL)	Restored *Lactobacillus* populations, reduced the Enterobacteriaceae abundance, and reduced muscle atrophy	2016	^147^
	*Lactobacillus paracasei*	SAMP8 mice (28-week-old)	18	Intragastric administration (1 × 10^9^ CFU/mouse/day)	Reduced oxidative stress, enhanced mitochondrial function and muscle strength, and delayed sarcopenia pathogenesis	2019	^136^
	*Lactobacillus casei, Bifidobacterium longum*	C57BL/6 mice (40-week-old)	46	Oral (2 × 10 ^9^ CFU/day)	Reduced inflammation and oxidative stress in peripheral tissues, improved intestinal barrier function, and enhanced muscle strength and mass	2019	^138^
	*Bifidobacterium longum*	ICR mice (8-week-old)	40	Oral (1 × 10^8^ or 10^9^ CFU/day)	Enhanced the muscle endurance, grip strength, and anti-fatigue potential of mice and improved the exercise performance of mice	2019	^206^
	*Lactobacillus plantarum*	Athletes and amateurs	34	Oral (1 × 10^10^ CFU/day)	Reduced inflammation and oxidation and improved muscle mass and exercise performance	2019	^207^
	GF	C57BL/6J mice (6–8-week-old)	42	Gavage of 100 μL homogenate generated from two fecal pellets of PF mice	GF mice developed muscle atrophy; after transplantation of the gut microbiota of healthy mice to GF mice, the GF mice showed increased skeletal muscle mass and decreased muscle atrophy marker expression	2019	^26^
	*Lactobacillus salivarius subspecies*	ICR mice (6-week-old)	40	Gavaged (1 × 10 ^9^ or 1 × 10^10^ CFU/day)	Increased the *L. reuteri* abundance in the intestinal tract of mice, promoted glycogen storage, and improved muscle strength and endurance	2020	^208^
	*Bifidobacterium breve*	SD rats, C57BL/6J mice (8-week-old)	86	Oral (1 × 10 ^9^ CFU/day)	Enhanced mitochondrial function, increased muscle mass and strength, and improved fatigue and muscle atrophy	2020	^209^
Bone and joint	*Bifidobacterium longum*	Sprague – Dawley rats (10-week-old)	24	Gavaged (1 × 10 ^8^ −1 × 10^9^ CFU/mL)	Regulated the abundance of osteoclasts and osteoblasts, promoted bone formation, inhibited bone resorption, altered the microstructure of the femur, and enhanced bone mineral density	2015	^180^
	*Lactobacillus reuteri*	C57BL/6 mice (14-week-old)	40	Gavaged (1 × 10^9^ CFU/mL)	Inhibited bone loss and promoted bone formation in type I diabetic mice by activating the anabolic pathway	2015	^210^
	*Lactobacillus rhamnosus*	C57BL/6J mice (10-week-old)	60	Gavaged (1 × 10^9^ total bacteria)	Prevented bone loss by enhancing the intestinal barrier integrity, thereby limiting osteoclast production	2016	^211^
	*Lactobacillus helveticus*	Sprague–Dawley rats (10-week-old)	24	Gavaged (1 × 10 ^8^-10^9^ CFU/mL)	Increased bone formation and prevented bone loss by increasing the expression of osteoblast genes	2018	^212^
	*Lactobacillus reuteri*	75–80-year-old women with low bone mineral density	70	Oral (1 × 10 ^10^ CFUs)	Reduced the loss of total tibial volume bone mineral density (BMD) in elderly women with low BMD	2018	^176^
	*Bacillus clausii*	BALB/c mice (8–10-week-old)	30	Added to drinking water (1 × 10 ^9^ CFU/mL)	Decreased Th17 cell abundance and proinflammatory cytokine (IL-6, IL-17, IFN-γ, and TNF-α) expression, increased anti-inflammatory cytokine (IL-10) expression, and enhanced bone health in postmenopausal osteoporosis mouse models	2018	^179^
	*Lactobacillus reuteri*	BALB/c mice (11-week-old)	46	Added to drinking water (3.3 × 10^8^ CFU/mL)	Changed the *Firmicutes* to *Bacteroidetes* abundance ratio, enhanced the intestinal barrier function, and prevented trabecular bone loss in the femur and vertebrae of mice	2019	^175^
	*Lactobacillus* *Lactococcus*	SAMP6 mice, SAMR1 mice (16-week-old)	40	Oral (1 × 10^8^ CFU/mL)	Improved osteoporotic conditions	2019	^174^
	*Bacteroides vulgatus*	C57BL/6 mice (6-week-old)	16	Gavaged (5 × 10^9^ CFU/mL)	Alleviated colonic microbiota dysregulation and downregulated colonic LPS/TLR-4/P-NF-κB signaling and serum TNF-α expression, thereby preventing lumbar bone loss in ovariectomized mice	2021	^177^
	*Lactobacillus casei*	Patients with rheumatoid arthritis	46	Oral (1 × 10^8^ CFU/day)	Lowered the serum hs-CRP levels, lowered proinflammatory factor expression, and reduced joint pain and swelling	2014	^129^
	*Lactobacillus casei*	Patients with knee osteoarthritis	537	Intake of milk containing *Lactobacillus casei* (6 × 10^9^ CFU/day)	knee osteoarthritis index and pain score by reducing the serum hs-CRP levels to treat joint disease	2017	^197^
	*Lactobacillus casei*	Sprague – Dawley rats	21	Oral (2 × 10^8^ CFU/day)	Protected AIA rats from bone destruction by regulating the dysregulation of the microbiota, and improved rheumatoid arthritis by reducing inflammation and oxidative stress	2019	^201^

This table shows the results of recent animal experiments and clinical experiments of the effects of intestinal flora on skeletal muscle, bone and joints. AIA: Adjuvant induced arthritis; CFU: Colony-forming unit; GF: Germ- free; PF: Pathogen- free; hs-CRP: hypersensitive-c-reactive-protein; IFN-γ: Interferon gamma.

## Effective intervention of musculoskeletal health based on intestinal microbiota

**Figure 2.**
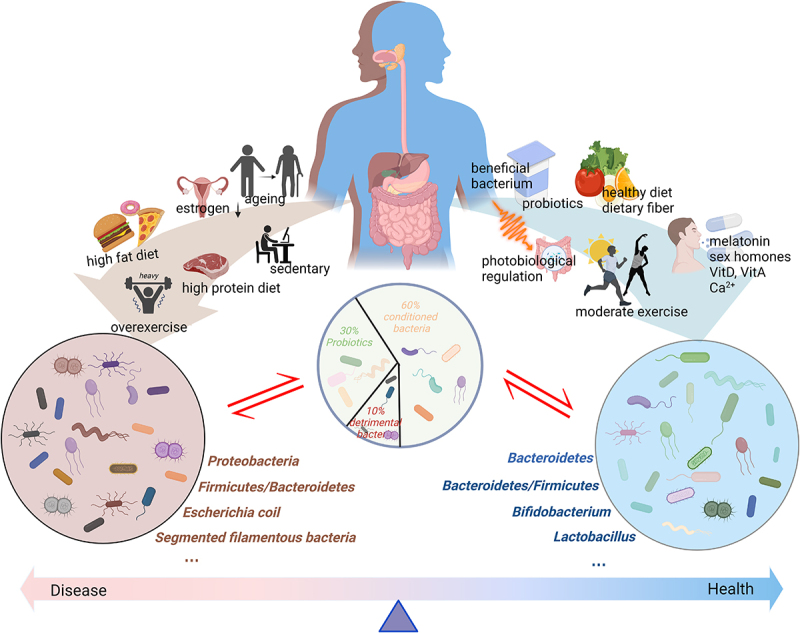
The intestinal microbiota is generally divided into three categories: probiotic, detrimental bacteria, and conditioned bacteria. Different lifestyles exert varied effects on the intestinal microbiota. Aging, the decreased secretion of sex hormones, high-fat diet intake, high-protein diet intake, and a sedentary lifestyle or over exercising can exert negative impacts on the intestinal microbiota. These cause an increase in the proportion of detrimental bacteria, inducing mucosal permeability and triggering a series of negative reactions. Meanwhile, a healthy diet, moderate exercise, photobiological regulation, supplementation with prebiotics and probiotics, vitamin intake, Ca^2+^, sex hormones, and melatonin can promote positive changes in the intestinal microbiota, thereby enhancing musculoskeletal system function. Created with BioRender.com.

Intestinal microbiota is closely related to the normal metabolism of the musculoskeletal system. Appropriate interventions can be adopted to promote the optimal regulatory role of intestinal probiotics (Figure 2).

### Supplementation with prebiotics, vitamins D and dietary calcium promotes musculoskeletal system health

Prebiotics are nutrients that are degraded by intestinal flora and support symbiote survival.^213^ Prebiotics are indigestible dietary fibers that are generally considered a type of dietary fiber. The intestinal microbiota can utilize prebiotics to rebuild the bacteria community.^139^ These provide beneficial physiological effects on the host musculoskeletal system by selectively stimulating the activity or growth of certain intestinal microbial strains. *Lactobacillus* and *Bifidobacterium* are common target bacteria for prebiotics, and inulin and oligosaccharides have been widely studied as prebiotics.^189^ Prebiotics improve intestinal barrier function and host immunity and reduce the abundance of potentially pathogenic bacteria such as *Clostridium*. Prebiotic supplements have been shown to promote positive changes in the skeletal muscles. Delzenne et al. showed that circulating LPS levels and inflammation decreased and muscle mass increased in mice fed prebiotic fiber oligosaccharides.^214^ In addition, prebiotic supplementation improved *Bifidobacterium* and *Lactobacillus* levels and prebiotic supplementation also affected the proportion of *Bacteroidetes* and *Firmicutes*.^215^ Concurrently, prebiotics, as a type of dietary fiber, can also promote the generation of SCFAs.^189^

Vitamin D is a key factor in increasing bone mass. Besides directly affecting calcium absorption, vitamin D also regulates intestinal mucosal homeostasis by maintaining the integrity of the intestinal barrier, which affects immune system function and inflammatory responses.^216,217^ Vitamin D improves homeostasis of the intestinal microbiome and facilitates muscle anabolism^218^. Vitamin D can inhibit the levels of pro-inflammatory factors and promote the anti-inflammatory factors secretion of body.^219,220^ Findings from specific studies suggest that vitamin D supplements can regulate gut flora and increase its diversity in women. In addition to the *Bacteroidetes* to *Firmicutes* ratio, the relative abundances of *Akkermansia* and *Bifidobacterium* were increased in response to vitamin D supplementation.^221^ Vitamin A has been shown to exhibit a similar function in maintaining the intestinal barrier function and regulating immune responses and bacterial diversity to maintain intestinal homeostasis.^222^ It has also been reported that vitamin D supplementation did not improve any sarcopenia index in older people in the community and may impair some aspects of bodily function. More experimental evidence is needed to clarify the role of Vitamin D.^223^ Therefore, the evidence from clinical trials needs to be strengthened.

Calcium is the most common mineral in the body, and its intake is related to the healthy development of bones. Calcium is also the most important element in maintaining the normal excitatory and contractile functions of skeletal muscles. Dietary calcium intake can cause changes in the gut microbiota. For example, mice fed with dietary calcium showed a significant increase in intestinal microbiota diversity and higher levels of *Bifidobacterium*, *Bacteroides*, *Ruminococcaceae* and *Akkermansia*.^224,225^ In addition, dietary calcium appears to have a protective effect on the gut barrier, increasing buffering capacity and promoting bone mass by increasing the absorption of dietary calcium in the host body.^224^ In summary, dietary calcium regulates the intestinal microbiota, establishes cross-dialogue with the host, promotes metabolism, and promotes musculoskeletal health.

### Diet regulates intestinal microbiota homeostasis and promotes musculoskeletal health

The intestinal microbiota has recently been defined as a “sensor” of host nutritional signals. The type and diversity of food consumed are closely associated with the composition of gut microbiota.^226^ Evidence from previous studies has shown that long-term changes in dietary patterns can induce changes in intestinal flora.^227^ Short-term dietary modifications can also lead to variations in the gut microbiota. For example, after switching from a plant-based diet to an animal-based diet, the concentrations of acetate and butyrate in the intestinal tract decreases significantly.^228^ Additionally, an animal-based diet increased *Bacteroidetes* and decreased *Firmicutes* by.^229^

Skeletal muscle mass is affected by muscle protein synthesis and breakdown, and changes in the intestinal microbiota with age are also affected by dietary protein intake.^230^ In addition, the intake of a protein-rich diet is positively correlated with the diversity of gut microbiota and can improve the abundance of *Bacteroidetes* in the gut microbiota.^231^ Notably, long-term high-protein diets do not always exert positive effects on muscles, and the long-term administration of beef protein supplements to endurance athletes was shown to decrease the abundance of beneficial intestinal bacteria such as *Bifidobacteria*.^232^ Moreover, compounds produced by fermentation of undigested protein residues in the colon exert potential negative effects on intestinal, immune and metabolic functions.^233^

Studies of high-fat diet mice have shown that the diet increases body weight and inflammatory marker expression and decreases glucose tolerance;^234^ more importantly, the circulating levels of LPS in high-fat diet-fed mice increased by two or three times, which led to an increase in intestinal permeability, thus triggering an inflammatory response that damages muscle mass.^235^ High-fat diet obesity caused by intake may also lead to excessive production of ROS and oxidative stress responses in the body and an accompanying increase in the adipokine population and TNF-α expression, thus increasing the chronic inflammatory response in the body and affecting skeletal muscle function.^236^ Moreover, high-fat diets can reduce SCFAs production and increase the proportion of Proteobacteria.^229^

In addition, higher carbohydrate intake was closely associated with a decreased diversity of intestinal flora. Although *Bifidobacterium* content increased, *Lactobacillus* and *Streptococcus* contents decreased. Coffee, tea and red wine are rich in polyphenols, which are associated with prebiotic abundance and *Bifidobacterium* activity.^237^ Dietary polyphenols can increase the bloom of SCFA-producing bacteria and inhibit the growth of LPS-producing bacteria, thus regulating the gut microbiota^238^ and affecting musculoskeletal system health. The intake of a Mediterranean diet helps maintain healthy gut flora because the diet incorporates a balanced intake of high-quality protein and complex carbohydrates and higher levels of fiber and polyphenols.^20^ Therefore, a healthy diet can improve the relative proportion of probiotics and regulate inflammation, whereas an unhealthy diet can lead to dysregulation of the intestinal flora, oxidative stress, inflammation, and other adverse effects, ultimately damaging the health of the musculoskeletal system.

### Positive effects of exercise on the intestinal microbiota promote musculoskeletal health

Moderate exercise can also improve the muscles, bones and joints. Our findings showed that exercise intervention can effectively improve muscle mass and function in elderly people aged >60 years, and the effect of exercise is significantly greater than that of nutritional supplements.^239^ Exercise can also effectively increase the mineral density of bone, improve bone strength, and reduce the risk of bone mass loss and incidence of falls and fractures.^240^ Similarly, exercise can relieve pain, enhance joint function, and improve the quality of life of people with osteoarthritis.^241^

Exercise can also improve musculoskeletal health by improving the intestinal microbiota. Exercise has been found to promote positive changes in intestinal microbiota, thus improving musculoskeletal function in both human and animal experiments. High-intensity training prevented obesity-related intestinal microbiome dysregulation and maintained intestinal microbiota diversity in high fat diet-induced obese mice. The *Bacteroides* to *Firmicutes* ratio in non-obese mice also changes after exercise, and the abundance of *Bacteroides* increases significantly after exercise.^242^ In a study on exercise and diet in obese rats, exercise exerted a stronger and more stable effect on the intestinal microbiota over time and could more effectively promote intestinal mucosal integrity and metabolic function.^243^

Similar results have been obtained from human studies on the relationship between exercise and gut microbiota. The relative abundance of the gut microbiota was significantly higher in elite athletes who underwent intensive training than in sedentary adults.^231^ Professional rugby players have a higher diversity of intestinal microbiota than nonprofessional athletes, and the intestinal microbiota of professional athletes primarily includes 22 phyla of bacteria, whereas the low body mass index (BMI) and high-BMI control groups only have 11 and 9 phyla of bacteria, respectively. Professional athletes also had lower levels of inflammatory cytokines than nonprofessional athletes.^244^ Additionally, research has shown that the intestinal microbiota (*Villanella*) can also affect athletic performance by converting lactic acid produced during exercise into propionic acid to prolong running time and improve athletic performance.^245^

The equilibrium between *Bacteroidetes* and *Firmicutes* in humans during aerobic exercise is critical for health maintenance, and disruption of the balance in bacterial colonization in the gut can lead to inflammation and metabolic or neurological diseases.^246^ A Japanese study of aerobic exercise intervention in the intestinal microbiota of older women showed that a 12-week aerobic exercise program not only increased the relative abundance of *Bacteroides* but also improved cardiorespiratory health. Concurrently, the abundance of *Bacteroides* was remarkably higher in subjects who increased their brisk walking time by >20 min.^247^ Therefore, moderate exercise can enhance musculoskeletal function by improving the composition of the gut microbiota, enhancing gut mucosal function, suppressing inflammatory responses, and maintaining a variety of intestinal flora.^248^

The potential effects of exercise on gut microbiota mediate the process of osteoarthritis.^249^ In an animal study, researchers administered rats with a high-sugar, high-fat diet along with aerobic exercise, prebiotics, and a combination of both and found that the combination of the two interventions completely prevented knee injury in obese rats.^250^ Interestingly, another study showed that a combination of aerobic exercise and prebiotics improved metabolic disorders in obese rats but did not improve preexisting osteoarthritis damage in the knee.^251^ Therefore, further research is required for a thorough investigation.

Excessive exercise may limit muscle formation by promoting inflammation and nutritional limitations as well as oxidative and metabolic stress. Other negative effects of overtraining include intestinal ischemia, increased intestinal barrier permeability, and oxidative stress, which lead to an imbalance in the intestinal microecology, an increase in inflammatory responses, increased catabolism, and deterioration of muscle function.^252^ Regular training appears to be associated with better biodiversity and beneficial effects on intestinal microbiota.^253^ Evidence from certain studies supports the idea that training for exhaustion may be linked to harmful microbial consequences.^254,255^ Therefore, the influence of exercise on the intestinal flora may depend on the intensity and duration of exercise.

### The effects of supplementation with estrogen and melatonin on the intestinal microbiota promotes musculoskeletal health

The ratio of gut bacteria to human cells differs between genders, with women having a higher ratio than men. The ratio of bacteria to human cells was 1.3 for men and 2.2 for women^256^. Women also have a higher diversity of gut microbes. *Akkermansia muciniphila* is especially abundant in females. Premenopausal women had a higher ratio of *Firmicutes/Bacteroidetes*, and relatively more abundant *Lachnospira* and *Roseburia* than postmenopausal women. The relative abundance of *Prevotella*, *Parasubacter* and *Bilophila* were lower in premenopausal women than in postmenopausal women, as well as lower IL-6 and monocyte chemoattractant protein-1 plasma levels, which represent inflammation levels.^257^ This suggests that estrogen may affect the regulation of intestinal microbial homeostasis and immunity.^258^ Conversely, an imbalance in the intestinal flora also affects estrogen activity. Reportedly, a significant decline in estrogen levels after menopause can cause damage to the gut barrier and bone health.^216^ As previously mentioned, the gut microbiota regulates estrogen by secreting β-glucosidase, and when this process is impaired by dysregulation of the gut flora (which is characterized by reduced microbial multiformity), it results in a decrease in circulating estrogen, which affects bone metabolism.^113^ It has been reported that non-ovarian estrogen is influenced more by the gut microbiome,^259^ which may be one of the reasons why postmenopausal women are more prone to OP. Therefore, the maintenance of intestinal homeostasis is essential for the normal secretion of estrogen and the balance of bone metabolism. Although the interaction between estrogen and intestinal microbiota has not been fully described, it is strongly suggested that the intervention of estrogen in intestinal microbiota may promote musculoskeletal health; however, direct evidence is still needed.

A recent review provides the latest evidence on how sleep disorders are associated with and contribute to changes in gut microbiome composition across the life cycle, offering an insight into the causes of sarcopenia through sleep disorders. It is thought that the loss of musculoskeletal health is related to sleep disorders. Meanwhile, in the older adults, shorter sleep duration was associated with an increase in pro-inflammatory bacteria, whereas improved sleep quality was positively correlated with an increase in beneficial *Warts microbacteria* and *Flatcoccus*. In young adults, the effect of sleep disruption on gut microbial composition, particularly the ratio of beneficial *Firmicute*s to *Bacteroidetes*, remains paradoxical and unclear. This study would not only deepen the understanding of the diverse contributing factors to sarcopenia but also offer a more comprehensive view of this complex condition. When sleep is poor, gut microbiome composition often changes, which may mediate the pro-inflammatory state between sleep disorders and sarcopenia.^260^ These findings not only suggested the important role of gut microbiota in the correlation between sleep quality and sarcopenia, but also implied that hormones regulating sleep, such as melatonin, may be one of the effective intervention targets.

Melatonin is another endogenous hormone that regulates sleep and circadian rhythms and exhibits antiaging, anti-inflammatory and antioxidant properties. Recently, it has been found to be a safe dietary supplement for disease treatment and skeletal muscle quality improvement.^261^ Melatonin reduces oxidative stress and inflammation and protects skeletal muscles from oxidative damage.^262^ Additionally, it can improve muscle mitochondrial function during aging.^263^ Studies in the elderly have also shown a significant correlation between melatonin levels and muscle strength.^264^ Interestingly, SD mice displayed weakened intestinal flora and a limited number of probiotic species, such as *Akkermansia*, *Bacteroides* and *Faecalibacterium*.^265^ Interestingly, melatonin treatment reversed this abnormal microbiome composition. Melatonin has also recently been shown to improve the intestinal microbiota in animals and humans. Oral melatonin supplementation can reduce lipid accumulation, reverse gut microbiota imbalance, and improve the diversity of the intestinal flora. SCFAs levels were significantly lowered in the intestinal tract of high fat diet-fed mice but were restored after melatonin supplementation.^266^ In addition, melatonin supplementation in high-fat diet-fed mice can effectively improve the intestinal ecological imbalance, and melatonin can change the *Firmicutes* to *Bacteroidetes* ratio and enhance intestinal mucosal function in obese mice. Meanwhile, melatonin supplementation alleviated insulin resistance caused by low-grade inflammation and high-fat diet intake in mice.^267^ Melatonin has been shown to regulate insulin sensitivity^268^ and thus has a critical effect in maintaining glucose homeostasis and regulating glucose metabolism. Melatonin is involved in bone metabolism. Melatonin can also be used to treat inflammatory osteolysis via the inhibition of osteoclast formation through the activation of the nuclear factor erythroid 2-related factor 2 (Nrf2)/catalase signaling pathway.^269^ Therefore, we can infer that melatonin can enhance the function of the gut mucosa, improve lipid and glucose metabolism, and promote the generation of SCFAs by regulating the dysregulation of the intestinal microbiota, thus eventually enhancing the function of the musculoskeletal system.

### Photobiomodulation (PBM) of the intestinal microbiota promotes musculoskeletal health and other interventions

As a topical treatment, PBM is used clinically for various conditions, including muscle fatigue, joint and tendon inflammation and wound and fracture healing.^270^ Recently, a close relationship has been reported between PBM and intestinal microbiota. After PBM was administered via irradiation to the abdomen of healthy mice, the gut microbiota of mice changed significantly, and the diversity of intestinal flora also increased significantly. The effect was most pronounced in mice treated with red light three times a week, but not in mice subjected to a single red-light treatment^271^. NIR light exerted a more significant effect than red light. After NIR irradiation, the ratio of probiotics in the intestinal microbiota of mice increased significantly, and the treatment also regulated the abundance of bacteria related to the imbalance in intestinal microbiota; this effect could be attributed to the anti-inflammatory and redox signaling effects of PBM on the intestinal microbiota.^270^ Ultraviolet radiation has also been shown to affect the intestinal microbiota structure and function in rat models of bone loss.^272^ In addition to its anti-inflammatory effects, UV radiation may also regulate bone metabolism by inducing vitamin D synthesis and intestinal calcium absorption, thereby promoting bone formation, decreasing bone absorption, and enhancing bone mineral density.^273^ This evidence suggests that although there is not much direct evidence, PBM still shows a potential role in maintaining musculoskeletal system homeostasis by regulating the imbalance of intestinal flora. PBM has the potential to act as adjunct therapy (along with diet and exercise) to balance the microbiome and promote musculoskeletal health.

Lifestyle interventions including exercise, electroacupuncture, and supplementation with probiotics have a direct impact on the gut microbiome, altering its composition and function, which opens up an innovative avenue for new treatment opportunities for patients with a wide range of chronic diseases. Based on a large number of relevant literatures, recent studies have combed through the role of lifestyle intervention in regulating intestinal flora and thereby interfering with chronic pain, and found that direct evidence is still relatively less, but it is confirmed that these methods can improve pain and quality of life to a certain extent^274^.

## Future directions and clinical implications

The correlation between the modifying effects of the intestinal microbiota and musculoskeletal health is not well understood. In addition to the damage caused by the cascade of inflammation induced by endotoxins and oxidative stress, whether the intestinal flora will also secrete other bioactive substances, such as isoenzymes,^275^ to further interfere with the musculoskeletal health of the host had not been deeply explored. Moreover, the application of non-drug treatments, such as exercise intervention, to promote the health of the intestinal flora and musculoskeletal system, and the reasonable selection of exercise intensity and duration and other parameters need to be explored further. In addition, methods for identifying and targeting the key microbiota contributing to musculoskeletal health, improving the functions of these microbial strains and their metabolites, and constructing a clearer microbial-musculoskeletal system interaction model are essential for future investigations.^144^ Furthermore, the combined application of various reasonable intervention methods constitutes a meaningful exploration.

Recent systematic reviews have shown that most of the experimental results in animal trials support the direct improvement of musculoskeletal health through interventions such as nutritional regulation, probiotics supplementation, and fecal bacteria transplantation. The relevant mechanisms discussed in this paper are also confirmed. In contrast, clinical studies are currently few and the results obtained are not consistent, especially the lack of high-quality clinical studies^139^. The reason may be closely related to the difference of flora and individual differences of population.^144^ Personalized treatment for intestinal flora regulation is both difficult and promising for the treatment of musculoskeletal system diseases.

In the clinical studies of fecal transplantation, the existing meta-analysis summarizing the review of the therapeutic effect of fecal transplantation on 85 diseases found that the most studies were conducted in infectious diseases and intestinal diseases, and very few studies directly related to musculoskeletal health. However, the paper also clearly points out the therapeutic value and vision of fecal bacteria transplantation in chronic low-grade inflammation-related diseases (such as diabetes and obesity), which needs to be confirmed by more rigorous clinical studies with larger samples.^276^ This will bring hope for the treatment of musculoskeletal system diseases by fecal bacteria transplantation.

Nevertheless, the gut microbiota has a profound impact on musculoskeletal health. The current clinical application has just begun, considering the complexity of the microflora and individual differences, whether it is suitable for the screening of bacteria to promote musculoskeletal health, or individual treatment based on human fecal bacteria transplantation; Whether it is the means and methods of intestinal microecological intervention, or the effect of intestinal microecological intervention, it still needs a lot of clinical evidence to support, which needs joint efforts to achieve.

## Conclusion

In conclusion, the potential mechanisms by which the gut microbiota regulates musculoskeletal health include protein, energy, blood lipids, glucose metabolism, inflammation levels, neuromuscular connectivity, and mitochondrial function. The composition and metabolic variation of the intestinal microbiota may affect the functions of the musculoskeletal system. Imbalances in the intestinal microbiota increase the levels of pro-inflammatory factors, activate oxidative stress pathways, and reduce muscle mass, and affect bone formation and absorption. The positive effects of intestinal probiotics on the musculoskeletal system are primarily mediated through direct or indirect effects that promote muscle synthesis and balance in the bone metabolism. In addition, intestinal probiotics can reduce inflammation and oxidative stress.

Based on the mechanism of action of the intestinal microbiota in the musculoskeletal system, different interventions, such as probiotics, prebiotics, vitamins and dietary calcium, can be used to improve the composition and metabolism of the gut microbiota and enhance musculoskeletal system function. Lifestyle interventions including exercise have a direct impact on the gut microbiome, altering its composition and function, which opens up an innovative avenue for new treatment opportunities for patients with a wide range of chronic diseases. Recent studies have combed through the role of lifestyle intervention in regulating intestinal flora and found that direct evidence is still relatively less. Furthermore, emerging approaches, such as estrogen and melatonin supplementation, and photobiological regulation, have shown potential in the regulation of intestinal microbiota and promotion of musculoskeletal health, especially when used in combination. In the current mechanism and application research, although there is many evidence in animal experiments and clinical research, there is a lack of higher quality clinical research, especially applied research.

## List of acronyms

Acronym: The full name
AIA: Adjuvant induced arthritis
ATP: Adenosine triphosphate
atrogin-1: muscle atrophy F-box
BMI: Body mass index
CFU: Colony-forming units
GF: Germ-free
GLP-1: glucagon-like peptide 1
hs-CRP: hypersensitive-c-reactive-protein
IBD: Inflammatory bowel disease
IFN-γ: Interferon gamma
IGF-1: Insulin-like growth factor 1
IGF-1R: Insulin-like growth factor 1 receptor
IL-1: Interleukin 1
IL-2: Interleukin 2
IL-4: Interleukin 4
IL-6: Interleukin 6
IL-8: Interleukin 8
IL-10: Interleukin 10
IL-17: Interleukin 17
LPS: Lipopolysaccharide
mTOR: muscle target of rapamycin
mTORC1: muscle target of rapamycin complex 1
Murf-1: Muscle ring finger protein 1
MyoD: myogenic regulatory factors D
NIR: Near infrared
NF-κB: nuclear factor-κb
Nrf2: Nuclear factor erythroid 2-related factor 2
OA: Osteoarthritis
OBs: osteoclasts
OCLs: osteoclasts
OP: Osteoporosis
PBM: Photobiomodulation
PF: Pathogen- free
RA: Rheumatoid arthritis
RANKL: receptor activator of NF-κB ligand
RNA: Ribonucleic Acid
ROS: Reactive oxygen species
SCFA: Short-chain fatty acids
Th-17: T helper cell 17
TLR: Toll like receptor
TNF-α: Tumor necrosis factor alpha
UV: Ultraviolet
5-HT: 5-hydroxytryptamine
